# Microbial diversity across tea varieties and ecological niches: correlating tea polyphenol contents with stress resistance

**DOI:** 10.3389/fmicb.2024.1439630

**Published:** 2024-08-26

**Authors:** Su-hang Yao, Chi Zhou, Sai-jun Li, Yu-han Li, Cheng-wen Shen, Yu Tao, Xin Li

**Affiliations:** ^1^Hunan Vegetable Research Institute, Changsha, China; ^2^College of Horticulture, Hunan Agriculture University, Changsha, China; ^3^Tea Research Institute in Hunan Academy of Agricultural Sciences/National Small and Medium Leaf Tea Plant Germplasm Resource Nursery (Changsha) Hunan Branch, Changsha, China

**Keywords:** microbial community, tea varieties, tea polyphenol contents, rhizosphere microorganisms, leaf endophytic microorganisms, beneficial microorganisms

## Abstract

**Introduction:**

Microorganisms exhibit intricate interconnections with tea plants; however, despite the well-established role of microorganisms in crop growth and development, research on microbes within the tea plant remains insufficient, particularly regarding endophytic microorganisms.

**Methods:**

In this study, we collected samples of leaves and rhizosphere soils from ‘Zhuyeqi’, ‘Baojing Huangjincha#1’, ‘Baiye#1’, and ‘Jinxuan’ varieties planted.

**Results:**

Our analyses revealed significant variations in tea polyphenol contents among tea varieties, particularly with the ‘Zhuyeqi’ variety exhibiting higher levels of tea polyphenols (>20% contents). Microbiome studies have revealed that endophytic microbial community in tea plants exhibited higher host specificity compared to rhizospheric microbial community. Analyses of across-ecological niches of the microbial community associated with tea plants revealed that soil bacteria serve as a significant reservoir for endophytic bacteria in tea plants, *Bacillus* may play a crucial role in shaping the bacterial community across-ecological niche within the tea plants with higher tea polyphenol levels. In the aforementioned analyses, the microbial community of ‘Zhuyeqi’ exhibited a higher degree of host specificity for leaf endophytic microorganisms, the topological structure of the co-occurrence network is also more intricate, harboring a greater number of potential core microorganisms within its nodes. A closer examination was conducted on the microbial community of ‘Zhuyeqi’, further analyses of its endophytic bacteria indicated that its endophytic microbial community harbored a greater abundance of biomarkers, particularly among bacteria, and the enriched *Methylobacterium* and *Sphingomonas* in ‘Zhuyeqi’ may play distinct roles in disease resistance and drought resilience in tea plants.

**Conclusion:**

In summary, this study has shed light on the intricate relationships of tea plant varieties with their associated microbial communities, unveiling the importance of microorganisms and tea varieties with higher tea polyphenols, and offering valuable insights to the study of microorganisms and tea plants.

## 1 Introduction

Tea, a beverage cherished worldwide, has been the subject of extensive research due to its health benefits, including its antioxidant, antibacterial, and anti-inflammatory properties (Zhang et al., 2019; Hu et al., 2022). Central to these benefits are tea polyphenols, compounds that not only enhance the flavor profile of tea but also offer substantial health advantages, playing a pivotal role in the prevention of various diseases (Khan and Mukhtar, 2018; Zhang et al., 2019). The concentration of these tea polyphenols in tea leaves has thus become a focal point of scientific inquiry, due to their enhanced health-promoting potential.

Recent advancements in sequencing technology have shed light on the complex interactions between tea plants and microorganisms, revealing the potential of tea polyphenols to address agricultural challenges such as crop rotation in tea cultivation through their interaction with root microorganisms (Fan et al., 2022). This underscores the importance of tea polyphenols not only to human health but also to the ecosystem of the tea plants, particularly its surrounding microorganisms, thereby opening new avenues for research in agricultural science and pathogen control. However, despite the recognized impact of microorganisms on tea polyphenols, the specific role of tea polyphenols in relation to microorganisms, especially endosymbiotic microorganisms, remains insufficiently explored.

Furthermore, exploration of rhizosphere microorganisms has been acknowledged as a forthcoming research priority with potential agricultural benefits (Kumawat et al., 2022), to given an example, mitigating the perils tea plantations face including biotic and abiotic stresses (Muoki et al., 2020), while the study of endosymbiotic microorganisms within tea plantations remains limited and often lacks specificity regarding the role of different tea varieties with various contents of tea polyphenols in microbial composition, comparatively (Suryanarayanan, 2020; Zhang et al., 2023).

With greater emphasis, while research in other crops has suggested that beneficial microorganisms could enhance plant resilience in other crops (Bakker et al., 2012; Vinale and Sivasithamparam, 2020), the application of this strategy in tea cultivation remains underexplored. This presents a critical area for investigation, particularly the interplay between the chemical composition of tea plants and the promotion of beneficial microbial community, which could have significant implications for plant health and tea production. Understanding the interplay between tea polyphenols and beneficial microbial community could unlock new strategies for enhancing tea plant resilience, improving crop yields, and potentially enriching the health-promoting properties of tea. This gap highlights the need for targeted research to elucidate the intricate symbiotic relationships between tea plants and their associated microorganisms, with a focus on endosymbiotic varieties.

Here, we collected rhizosphere soils and tender leaves of different varieties of tea plants from the same tea plantation, and utilizing 16S rRNA gene and ITS amplicon sequencing techniques, we offer comprehensive analyses of microbial diversity across different tea varieties. This study seeks to unravel whether variations in tea polyphenol contents influence the composition of microbial community, the relationship between rhizosphere and endophytic microbes, and the implications for leveraging the tea plants with higher tea polyphenols in microbiological research. By elucidating the intricate relationships between tea polyphenols and microbial community, especially the tea plants with a higher content of tea polyphenols, specifically those where the polyphenol contents in the leaves exceeding 20%, our research aims to provide new insights into the complex interactions within the tea plantation ecosystem and contribute to the development of sustainable agricultural practices.

## 2 Materials and methods

### 2.1 Experimental design and sampling

In Hunan Province, China, at the Hunan Agricultural University Chang’an Internship Site in Changsha (28 °11′ N, 112 °58′ E), the rhizosphere soil and leaf samples of ‘Zhuyeqi’ (ZYQ), ‘Baojing Huangjinchayihao’ (HJC), ‘Baiye#1’ (AJBC), and ‘Jinxuan’ (JX) have been gathered in September 2023, and all of these tea plants belong to the species *Camellia sinensis*. The tea plantation has been cultivating these tea plants since 1989, and has adhering to conventional annual management practices for construction and fertilization.

For each variety, six tea plants were randomly selected for rhizosphere soil sampling, and four for leaf sampling. Soil samples were meticulously collected from approximately 15 cm depth near the absorbing roots. Leaf samples, the 1st to 3rd leaves from the tender or non-woody stems, underwent a 1-min pre-treatment with 1.0% NaClO to eliminate surface microorganisms. All samples were preserved in a foam box with dry ice during transportation and subsequently stored at −80°C for further analyses.

### 2.2 Tea polyphenol contents test

The determination of tea polyphenol contents was conducted using the Folin–Ciocalteu colorimetric method, as outlined in GB/T8313-2008.

Specifically, 0.2 g of uniformly ground tea leaf samples are weighed and placed in a 10 mL centrifuge tube. Subsequently, 5 mL of 70% methanol solution at 70°C is added, and the mixture is then subjected to extraction at 70°C in a water bath for 10 min, with stirring every 5 min. After the extraction is complete, allow it to cool to room temperature and then centrifuge at 3000 r/min for 10 min. Transfer the supernatant to a 100 mL volumetric flask, and repeat the process with the residue to ensure complete extraction of catechins. Combine the supernatants from the two extractions and make up the volume to 100 mL to obtain the test solution. Take 1 mL of the test solution, 5 mL of Folin-ciocaltcu reagent, and 4 mL of 7.5% Na_2_CO_3_ solution in a 10 mL test tube. After thorough mixing, let it stand for 1 h at room temperature, then measure the absorbance using a spectrophotometer and calculate the tea polyphenol contents according to the following formula.

Tea polyphenol contents (%) = $\frac{\left(C*E1*E2\right)}{G}*100\%\text{.}$

The components in the formula are as follows: C: Mass of catechins in the test solution as determined by absorbance (mg); E1: Volume of extraction solvent (mL), which is 10 in this experiment; E2: Dilution factor, which is 100 in this experiment; G: Dry weight of the sample (g), precise to 0.0001 g.

### 2.3 Microbial DNA extraction and amplification

The process of extracting microbial DNA was conducted using the TGuide S96 Magnetic Bead Kit, designed specifically for Soil/Fecal Genomic DNA Extraction. Post-extraction, the DNA samples were preserved at −20°C. The concentration of these samples was accurately measured using an enzyme marker provided by GeneCompang Limited (synergy HTX), ensuring the integrity of the samples was maintained throughout the process.

For the amplification phase, PCR products underwent electrophoresis in a 1.8% agarose gel, supplied by Beijing BBMF Technology Co., Ltd. The primer sequences employed for the amplification of bacteria and fungi DNA were chosen: for the 16S rRNA gene, the forward primer 515F (5′-GTGCCGCCGCGGGTAA-3′) and the reverse primer 806R (5′-GGACTACHVGGGTWTCTAAT-3′) were used; for the ITS region, the forward primer ITS1 (5’-CTTGTCATTTAGGAA-3′) and the reverse primer ITS2 (5′- GTGCGTTCTTCATCGATGC-3′) were selected.

The PCR reaction was prepared with a total volume of 10 μL, comprising 0.6 μL of F/R primer, a DNA sample ranging from 5 to 50 mg, 5 μL of KOD FX Neo Buffer, 2 μL of dNTPs (2 mM each), and 0.2 μL of KOD FX Neo, with the volume adjusted to 10 μL with ddH2O. The amplification protocol included an initial pre-denaturation at 98°C for 5 min, followed by 25 cycles of denaturation at 98°C for 30 s, annealing at 50°C for 30 s, and extension at 72°C for 40 s, with a final extension at 72°C for 7 min.

Post-amplification, the PCR product (10 μL) was combined with magnetic beads in a 1:1 ratio for fragment screening, and then eluted in 8–10 μL. The purification process involved mixing 10 μL of VAHTSTM DNA Clean Beads with the PCR product, followed by a series of washing steps with 80% ethanol and drying phases to ensure the removal of impurities. The purified DNA was then resuspended in 8–10 μL of ddH_2_O and subjected to quality control using the Qsep-400 method.

Samples that passed the quality control were subsequently sequenced on the Illumina NovaSeq6000 platform, adhering to the highest standards of genomic research and ensuring the reliability of the sequencing data for further analyses.

### 2.4 Sequence analyses

The initial phase of sequence analyses involved refining the quality of raw data through the application of Trimmomatic (version 0.33). Following this, Cutadapt (version 1.9.1) was used in identifying and excising primer sequences, further purifying the dataset. The USEARCH (version 10) was utilized in splicing double-ended reads and eradicating chimeras (UCHIME, version 8.1), culminating in the procurement of high-quality sequences ready for in-depth analyses.

Operational Taxonomic Units (OTUs) clustering was performed using USEARCH (version 10.0), which clustered sequences with a 97% similarity threshold. The selection of OTUs adhered to a stringent criterion, employing a default threshold of 0.005% of the total sequenced sequences. Amplified Sequence Variant (ASV) analyses was meticulously conducted with QIIME2 (version 2020.6), which denoised the quality-controlled data and filtered ASVs, setting a benchmark threshold of 0.005% of all sequences. This process was followed by the annotation of OTUs with QIIME2. Biomac Biotechnology Company has completed this portion and is responsible for the deposite of the raw data.

### 2.5 Inoculation of *Sphingomonas* inoculation and drought experiments on tea seedlings

The bacterium *Sphingomonas sanguinis*, utilized in this experiment, was isolated from the leaves of the tea plants by the Hunan Vegetable Research Institute. Specifically, 2 g of surface-sterilized tea leaves were placed in a 10 mL centrifuge tube with 10 mL of sterile water and grinding beads. They were then processed in a grinder at 480 Hz for 10 min. Using a pipette, 100 μL of the liquid from the ground centrifuge tube were evenly spread onto Luria-Bertani (LB) medium. The plates were then cultured at 25°C until single colonies appeared. Using an inoculation loop, these single colonies were separately transferred into 10 mL liquid LB medium, with each colony inoculated into two tubes of liquid medium, one tube of these tubes was sent to Beijing Qingke Biological Technology Co., Ltd. for identification, while the other was preserved at −80°C.

Initially cultured on LB medium, the strain was then transferred to 50 mL of liquid LB medium and incubated to obtain a viable bacterial concentration. The *Sphingomonas* fermentation liquid was applied to the leaves of Huangjin (*Camellia sinensis*), with sterile pure water used for the control group. Post-inoculation, both groups were maintained in a constant temperature incubator (28°C, 50% relative humidity, 7 d) followed by malondialdehyde (MDA) content determination. The MDA content was assessed using a microplate method, employing an MDA detection kit sourced from ABBKINE, following the manufacturer’s instructions.

### 2.6 Bioinformatics and statistical analyses

The core of the bioinformatics and statistical analyses was anchored in the utilization of the BMKCloud and R (v4.3.2) software packages. The Shannon diversity index, a measure of alpha diversity, was calculated at the OTU level, providing insights into the richness and evenness of microbial community. Beta diversity, evaluated through the Bray-Curtis distance metric, shed light on the structural variations in microbial community across different samples, as elucidated by previous analyses (Favere et al., 2020). Venn diagrams were generated to visualize the shared and unique OTUs among samples or groups using the R package “VennDiagram” (Jia et al., 2021). These findings were visualized via principal coordinate analyses (NMDS) and validation of ANOMISM computations (Marupakula et al., 2016).

The exploration of co-occurrence patterns was achieved by calculating multiple correlations and similarities within the network. A statistically robust correlation, defined by a Pearson correlation (r) greater than 0.7 and a *p*-value less than 0.05, indicated valid co-occurrence between taxa. This network analyses, where nodes represented individual genera and edges denoted pairwise correlations, highlighted significant metabolic associations and was visualized using the igraph package.

LEfSe (Linear Discriminant Analyses of Effect Sizes) and FUNGuild (FungiFunctionalGuild) were employed to discern taxa significantly differentiated between groups and to categorize fungi into ecologically meaningful categories, respectively, following the guidelines set by previous analyses (Segata et al., 2011; Nie et al., 2018). The investigation into the correlations between *Colletotrichum* and bacteria was facilitated by the corrplot package, with significant correlations identified under the same rigorous criteria. The distribution of bacterial genera across different tea plant varieties was depicted through species stacked plots, showcasing the top 10 bacterial counts and aggregating the rest under “others.” Furthermore, correlation analysis was conducted between screened beneficial microorganisms and the content of tea polyphenols, utilizing the “Pearson” method.

Comprehensive statistical analyses was conducted using SPSS 26.0 including performing analysis of variance (ANOVA) and significance analyses on the tea polyphenol contents and MDA contents. With the resultant data visualized through R, Gephi and GraphPad Prism 8, ensuring a clear and objective presentation of the experimental findings.

## 3 Results

### 3.1 The detection of tea polyphenol contents

**Figure 1:**
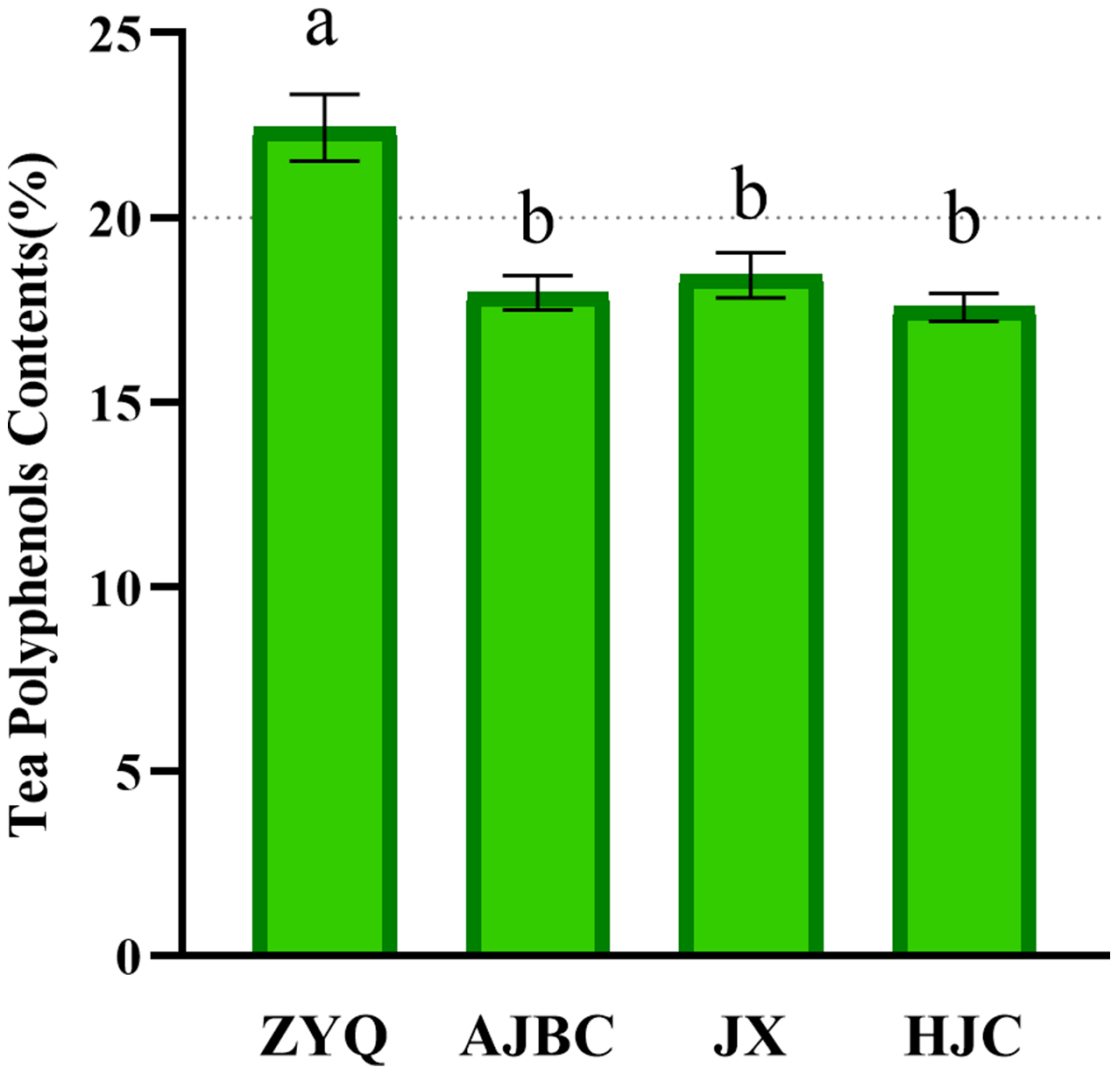
Tea polyphenol contents in leaves of different varieties of tea plants. Different lower case letters indicate significant differences (*p* < 0.05, LSD test).

Our investigation commenced with an analyses of the tea polyphenol contents in the leaves of various tea plant varieties, to recognize the critical role these compounds play in microbial community. The study revealed significant variations in polyphenols concentrations across the varieties examined. Notably, the tea polyphenol contents in the ZYQ exhibited significant higher compared to three other tea plant varieties. Specifically, the tea polyphenol contents in ZYQ’s leaves surpasses the 20% threshold, whereas the other three tea plant varieties fall below this mark. ZYQ’s tea polyphenol contents notably exceeded that of the other three tea plant varieties. (Figure 1; Supplementary Table S1). The subsequent analyses particularly focused on the microbial community characteristics of ZYQ.

### 3.2 Diversity analyses of microbial community structure

**Figure 2:**
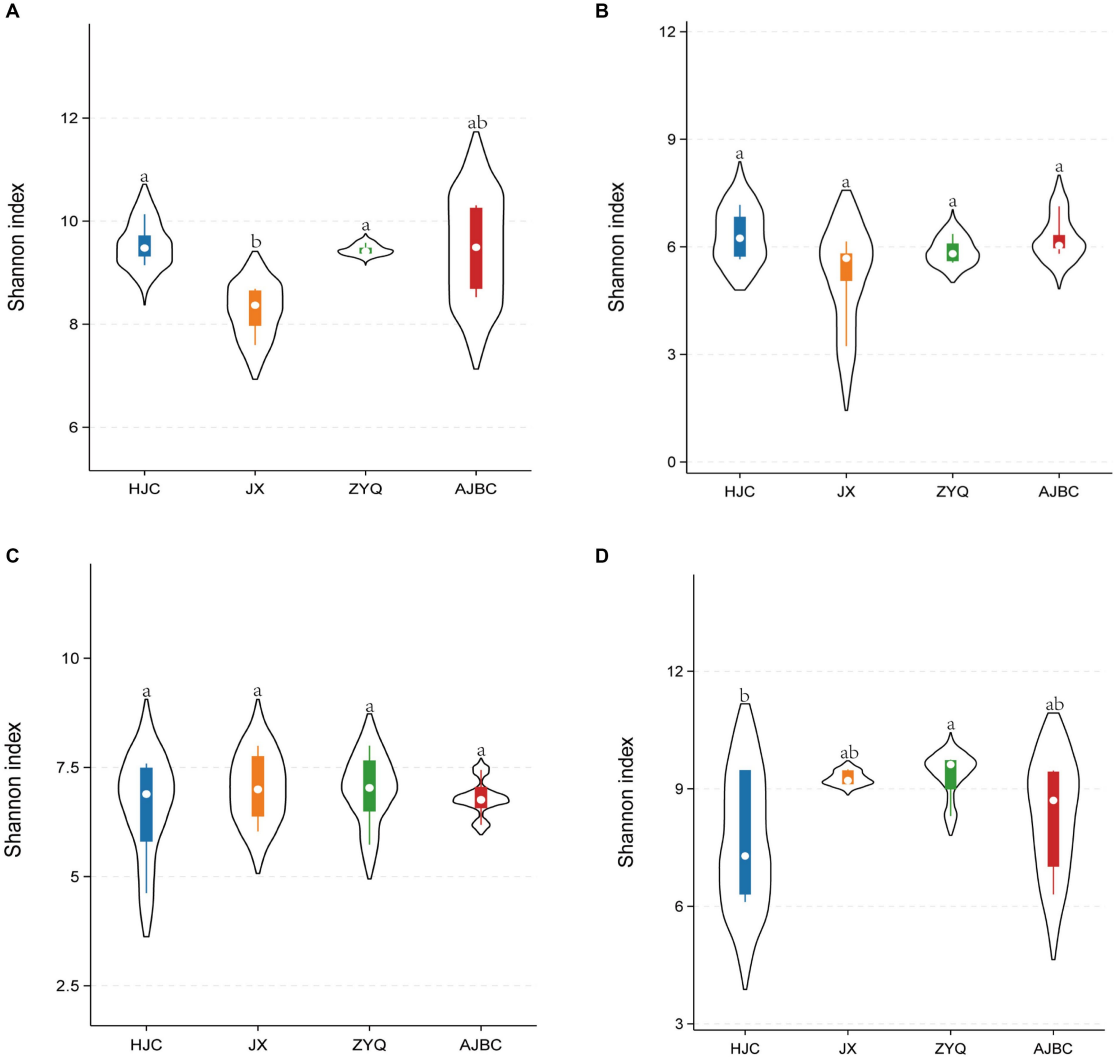
*α*-Diversity analyses (Shannon’s index). **(A)** Leaf-endophytic bacteria; **(B)** Leaf-endophytic fungi; **(C)** Rhizosphere bacteria; **(D)** Rhizosphere fungi Horizontal lines indicate intermediate bands. The top and bottom of the boxes represent the 75th and 25th quartiles, respectively. Thin lines represent 95% confidence intervals. Mean ± standard error; different letters indicate significant differences (*p* < 0.05) based on one-way ANOVA and LSD test.

To further our understanding of the microbial community structure associated with various tea varieties and their ecological niches, we conducted an analyses focusing on the *α*-diversity of microbial community (Figure 2). The Shannon index of bacterial community in the leaf blades of both HJC and ZYQ significantly exceeded that of JX; there is no significant difference observed in the fungal community within the leaves and rhizosphere bacterial community among the four tea plant varieties; however, concerning the rhizosphere fungal community, the Shannon index of HJC notably falls below that of ZYQ. Overall, the inter-group differences of the Shannon indices at the same niches do not exceed 2.

**Figure 3:**
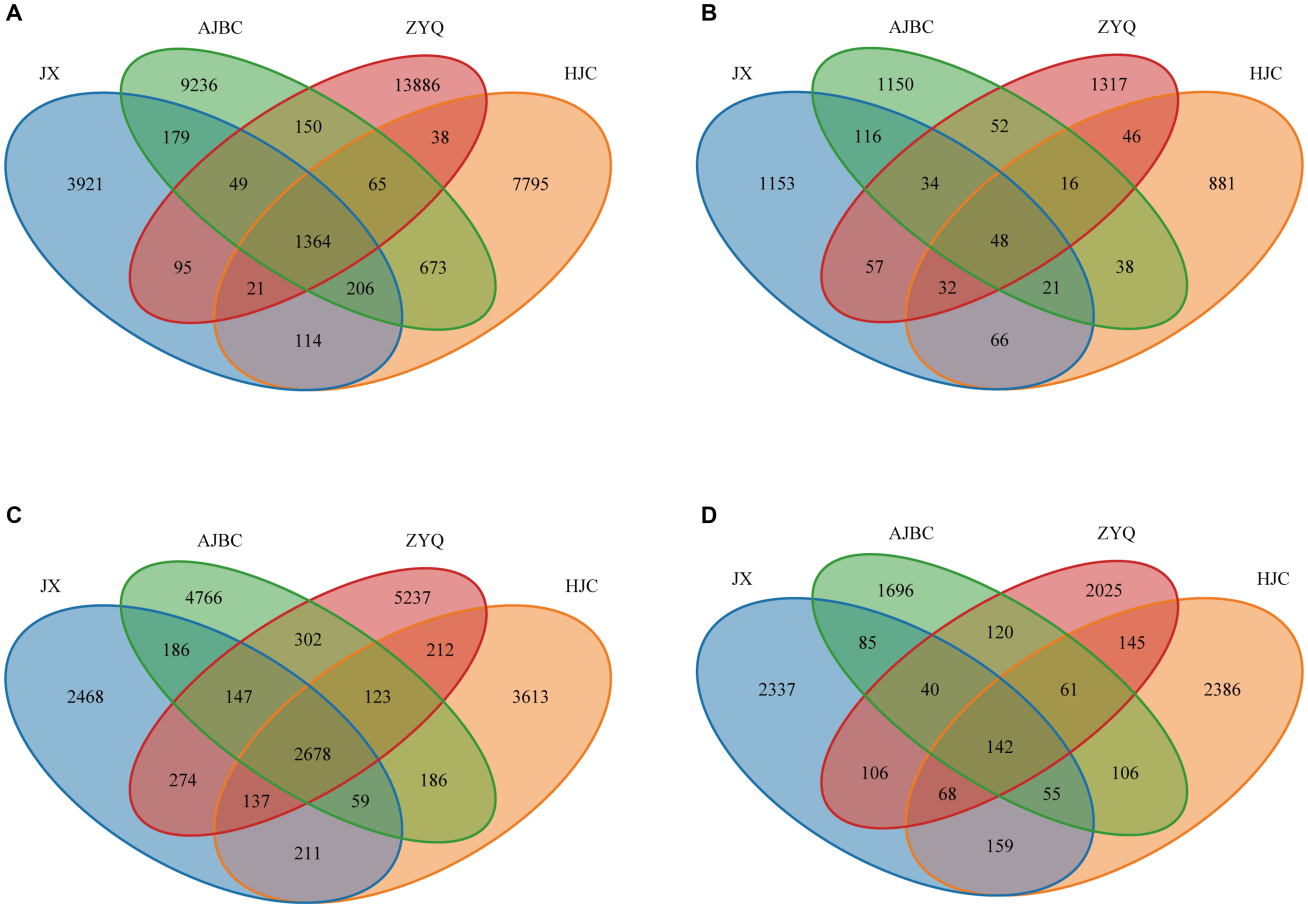
Venn diagrams. **(A)** Leaf endophytic bacterial community; **(B)** Leaf endophytic fungal community; **(C)** Rhizospheric bacterial community; **(D)** Rhizospheric fungal community.

To find out the influence of tea plant varieties and ecological niches on the tea microbial community, we analyzed the composition and structure of the microbial community (Figure 3). The shared OTUs of leaf endophytic bacterial community was 1,364, the unique OTUs of leaf endophytic bacterial community was JX (3921), AJBC (9236), ZYQ (13886), HJC (7795); The shared OTUs of leaf endophytic fungal community was 48, the unique OTUs of leaf endophytic fungal community was JX (1153), AJBC (1150), ZYQ (1317), HJC (881). As for rhizospheric microbial community, the shared OTUs of rhizospheric bacterial community was 2,678, the unique OTUs of rhizospheric bacterial community was JX (2468), AJBC (4766), ZYQ (5237), HJC (3613); The shared OTU of rhizospheric fungal community was 142, the unique OTUs of rhizospheric fungal community was JX (2337), AJBC (1696), ZYQ (2025), HJC (2386). In general, the unique OTUs of bacteria at the same ecological niche exceeds that of fungi, with the leaf endophytic microbiota possessing more unique OTUs than the rhizosphere. In addition, the unique bacterial OTUs of ZYQ vastly surpasses that of other 3 varieties, both in rhizosphere and within the leaf.

**Figure 4:**
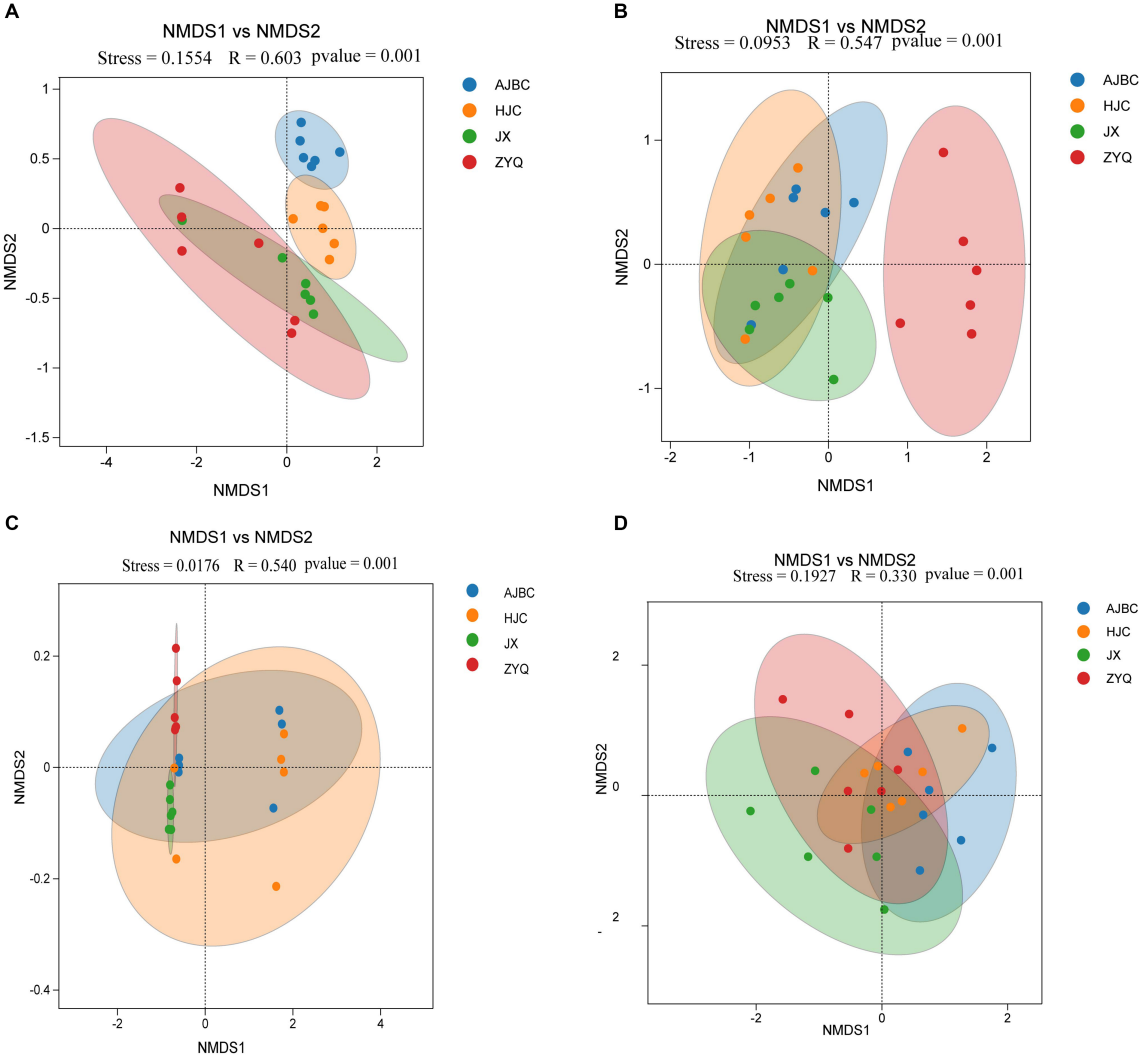
*β*-Diversity analyses (NMDS analyses). **(A)** Leaf endophytic bacteria; **(B)** Leaf endophytic fungi; **(C)** Rhizosphere bacteria; **(D)** Rhizosphere fungi.

The *β*-diversity of leaf endophytes was explored by using the Bray-Curtis distance (Figures 4A,B). All Stress values from NMDS analyses were below 0.2, indicating the reliability of the analytical outcomes. Specifically, the R-values for the ANOMISM computations of leaf endophytic bacteria and leaf endophytic fungi stood at 0.603 and 0.547, respectively (*p* < 0.05); as for rhizospheric bacteria and fungi stood at 0.540 and 0.330, respectively. This exploration revealed that the composition of the plant endophyte community was influenced by the specific tea varieties, including both bacterial and fungal populations. Intriguingly, the leaf endophytes of ZYQ, characterized by higher levels of tea polyphenols, demonstrated a relative separation from those associated with the other three tea varieties. In contrast, the leaf endophytes of the remaining varieties showed a more clustered pattern.

The structure of the rhizosphere microbial community was also found to be species-specific. Utilizing the Bray-Curtis distance to calculate *β*-diversity, the NMDS results highlighted discernible differences in bacterial and fungal community structures across the rhizospheres of different tea plants (Figures 4C,D). The R-values for rhizospheric bacteria and rhizospheric fungi were 0.54 and 0.33, respectively (*p* < 0.05), indicating a certain level of variation in the rhizosphere microbial community among different tea varieties. However, it’s important to note that these differences were not as pronounced as those observed among leaf endophytes, since the R-value for leaf endophytes is substantially higher than that of rhizospheric microbiota.

### 3.3 Co-occurrence network analyses of leaf-rhizosphere microbial community

**Figure 5:**
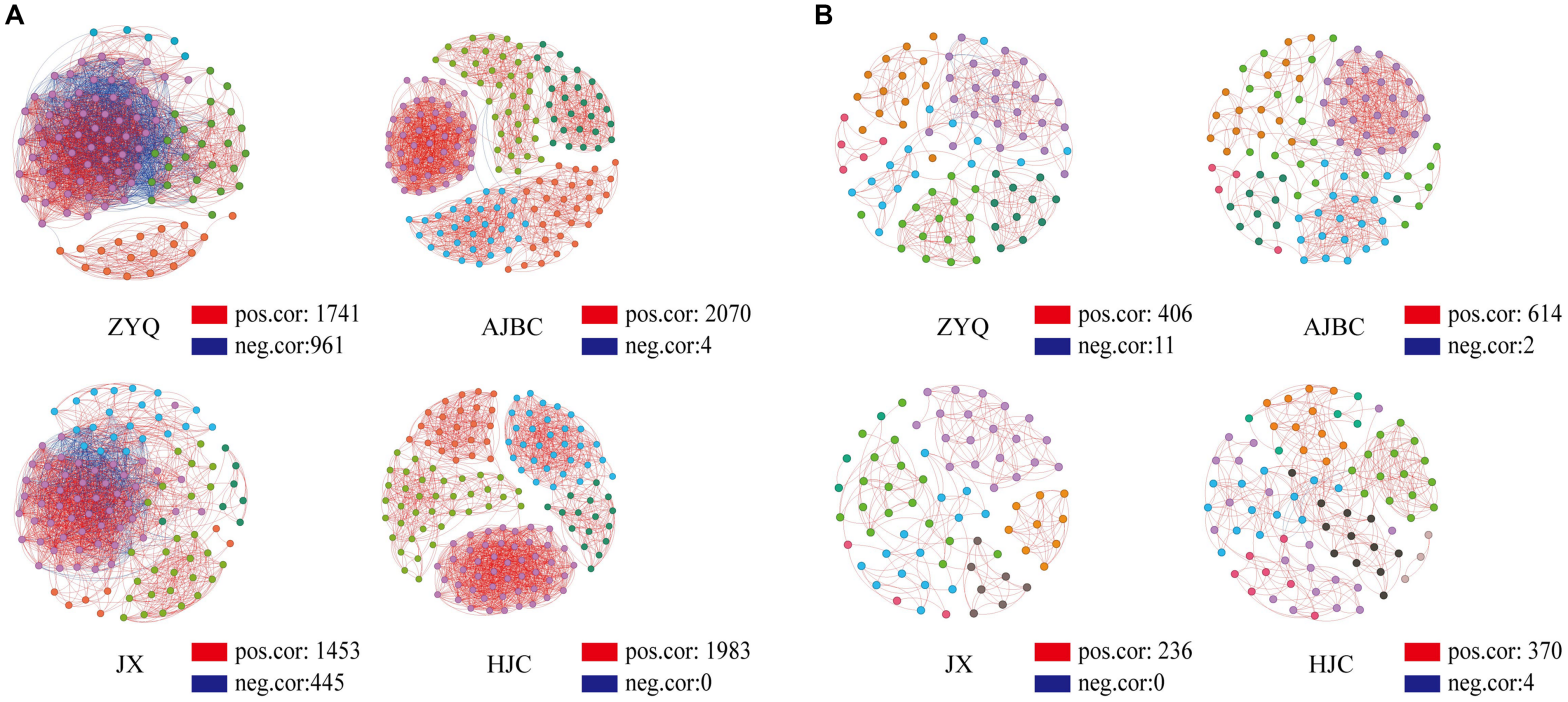
Co-occurrence network analyses. **(A)** Bacterial co-occurrence network based on genera correlation analyses. Genera with relative abundance above 0.05% were selected. One linkage represents strong (Pearson’s r > 0.7) and significant (*p* < 0.05) correlations. Red edges indicate positive correlations and blue edges indicate negative correlations; points of different colors indicate belonging to different modules in the co-occurrence network analyses. **(B)** Fungal co-occurrence network based on OTU correlation analyses, analyzed in the same way as in **(A)**.

The outcomes of the co-occurrence network analyses are illustrated in Figure 4. The interaction edges for the bacterial community within ZYQ, AJBC, JX, and HJC were quantified as 2,702, 2074, 1898, and 1983, respectively, The connectivity and average degree were as follows: 43.50% and 48.25, 15.70% and 33.00, 29.00% and 25.44, 15.70% and 25.24 (Figure 5A; Supplementary Table S2). For fungal community, the leaf-rhizosphere co-occurring edges in ZYQ, AJBC, JX, and HJC were 417, 616, 236, and 374, respectively, with connectivity rates of 11.4, 15, 8.9, and 10.5% and average degree were 9.69, 6.46, 12.96, 8.80, respectively (Figure 5B; Supplementary Table S3). This study observed that bacterial symbiotic edges surpassed those of fungi, indicating a more complex and intimate interactions within leaf-rhizosphere among bacteria. Notably, tea plants with higher levels of tea polyphenols, ZYQ, demonstrated significantly enhanced symbiotic edges and bacterial connectivity.

The topological properties of nodes within the network were further analyzed. Within the bacterial community, the average degree of nodes in the co-occurrence network for ZYQ, AJBC, JX, and HJC were found to be 48, 21, 30, and 21, respectively (Supplementary Table S4), furthermore, *Bacillus* has relatively high degree of connectivity in the co-occurrence networks of the four tea varieties (ZYQ: 56; AJBC: 2; JX: 24; HJC: 0). In the fungal community, the average degree of nodes in the co-occurrence network for ZYQ, AJBC, JX, and HJC were found to be 7, 11, 4, and 5 (Supplementary Table S5). The co-occurrence network of the bacterial community displays higher average degree, with ZYQ exhibiting a notably elevated average degree compared to the other three species. What’s intriguing is the discovery within ZYQ’s bacterial community, where *Methylobacterium*, *Sphingomonas*, and *Bacillus* exhibit higher degree of connectivity, The degree of these three genera in ZYQ, AJBC, JX, and HJC are respectively: ZYQ (67, 64, 56), AJBC (6, 0, 2), JX (0, 13, 24), HJC (0, 15, 0).

### 3.4 LEfSe analyses of leaf endophytic microbial community

**Figure 6:**
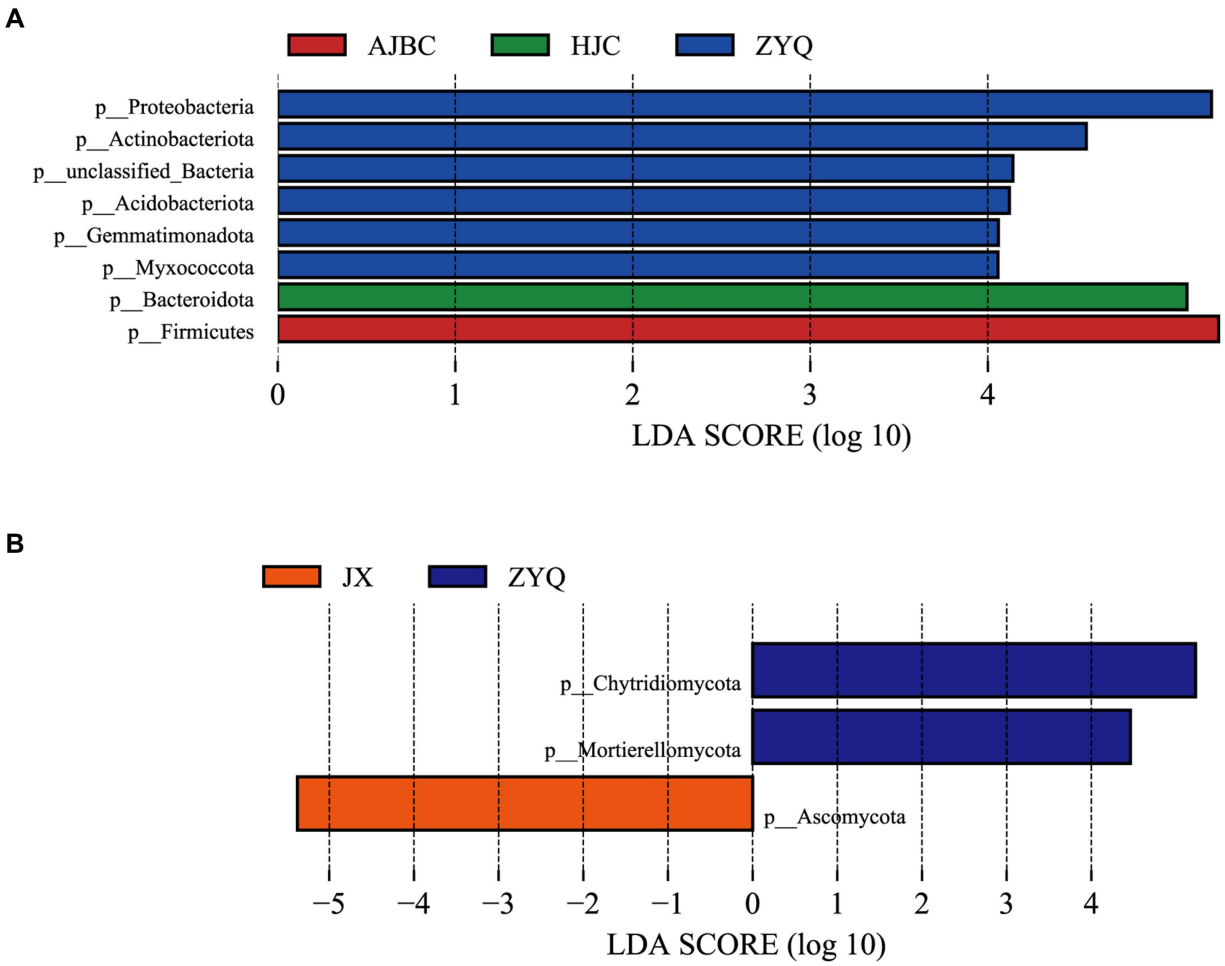
LEfSe analyses. **(A)** LEfSe results of different species of bacteria, different colors represent different species of different bacteria (*p* < 0.05, LDA > 2.0). **(B)** LEfSe results of different species of fungi, different colors represent different species of different fungi (*p* < 0.05, LDA > 2.0).

To thoroughly assess the beneficial microorganisms enriched by tea plants with higher tea polyphenol levels, separate LEfSe analyses for bacteria and fungi were conducted (Figure 6). The LEfSe analyses revealed 8 biomarkers in bacterial community (ZYQ: 2; AJBC: 0; HJC: 0; JX: 1), including Proteobacteria, Actinobacteriota, unclassified-Bacteria, Acidobacteriota. Gemmatimonadota, Myxococcota, Bacteroidota, Firmicutes, and 3 biomarkers in fungal community (ZYQ: 6; AJBC: 1; HJC: 1; JX: 0), including Chytridiomycota, Mortierellomycota, Ascomycota, respectively. This outcome suggests that the specificity of bacterial species within leaves is substantially higher than that of fungal species within leaves. Additionally, the number of bacterial biomarkers associated with ZYQ is significantly higher compared to the other three tea varieties.

### 3.5 *Methylobacterium* and *Sphingomonas*, which exhibit a significant positive correlation with tea polyphenol content, respectively confer advantages to tea plants in disease resistance and drought tolerance

**Figure 7:**
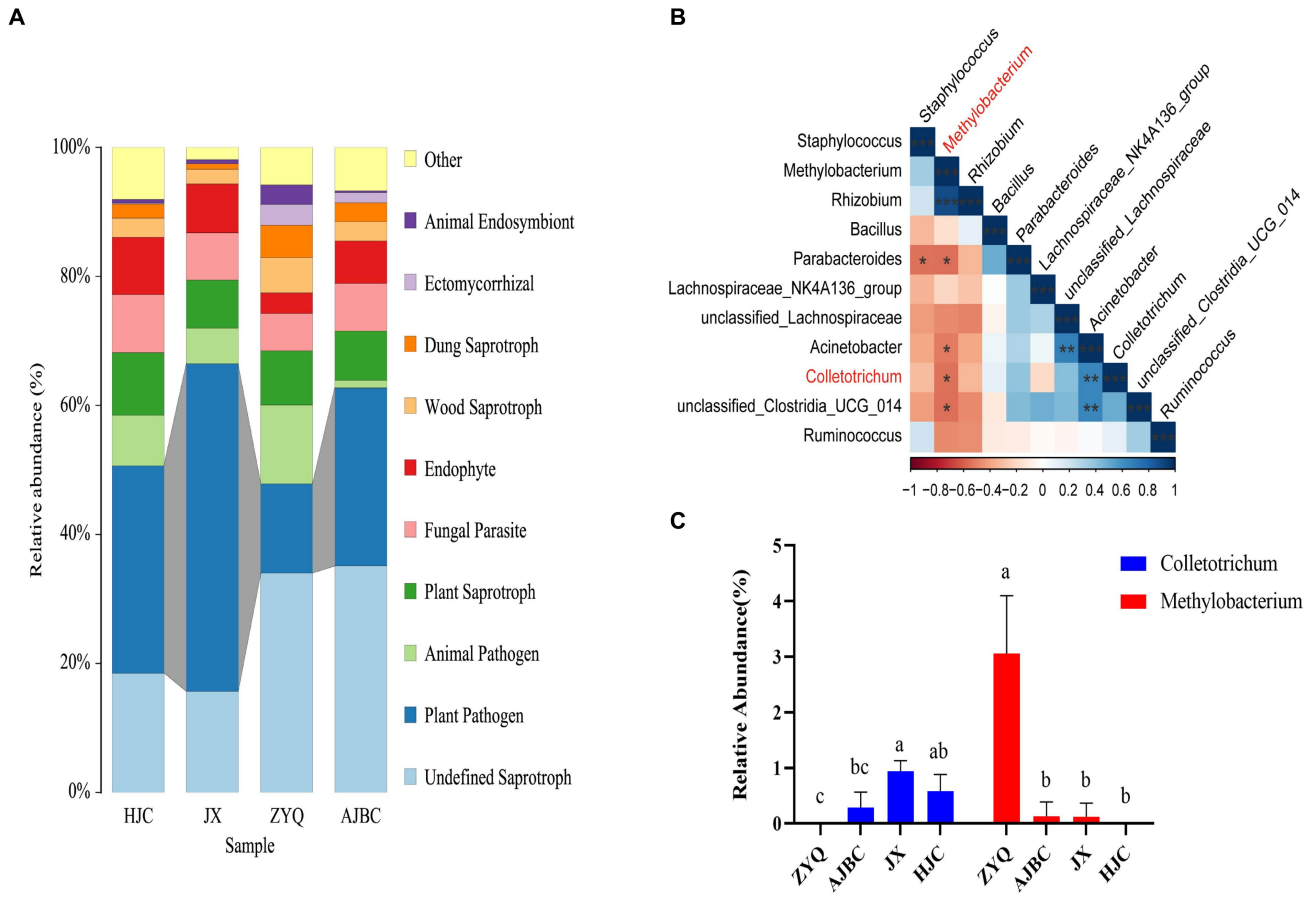
Screening of disease-resistant microorganisms in tea plants with higher levels of tea polyphenols. **(A)** FUNGuild prediction of leaf endophytic fungi of different varieties of tea plants. **(B)** Correlation between *Colletotrichum* and the top 10 leaf endophytic bacteria in relative abundance. Red color indicates negative correlation, blue color indicates positive correlation, and “*” indicates statistically significant correlation (*p* < 0.05). **(C)** Relative abundance of *Colletotrichum* and *Methylobacterium* in leaves of different varieties of tea plants. Different letters indicate significant differences (*p* < 0.05, LSD test).

Prior to identifying beneficial microorganisms in the tea plants with higher levels of tea polyphenols ZYQ, a FUNGuild prediction of leaf endophytic fungi was performed. The analyses indicated a significant reduction in plant pathogens in ZYQ compared to the other tea plants (Figure 7A; Supplementary Table S6).

In order to validate this hypothesis, we conducted a correlation analyses between the top 10 endophytic bacteria in relative abundance in leaves and *Colletotrichum* (Figure 7B). Further analyses indicated that, the relative abundance of *Methylobacterium* in ZYQ was notably higher compared to other tea varieties, with *Colletotrichum* undetected in ZYQ leaves (Figure 7C; Supplementary Table S7), supporting the hypothesis that enriched beneficial microorganisms in ZYQ contribute to pathogen resistance. The correlation analysis of tea polyphenol contents and the relative abundance of *Methylobacterium* revealed a significant positive association, suggested that the enriched tea polyphenols in ZYQ could be a potential factor contributing to the abundance of *Methylobacterium* (Supplementary Table S8).

**Figure 8:**
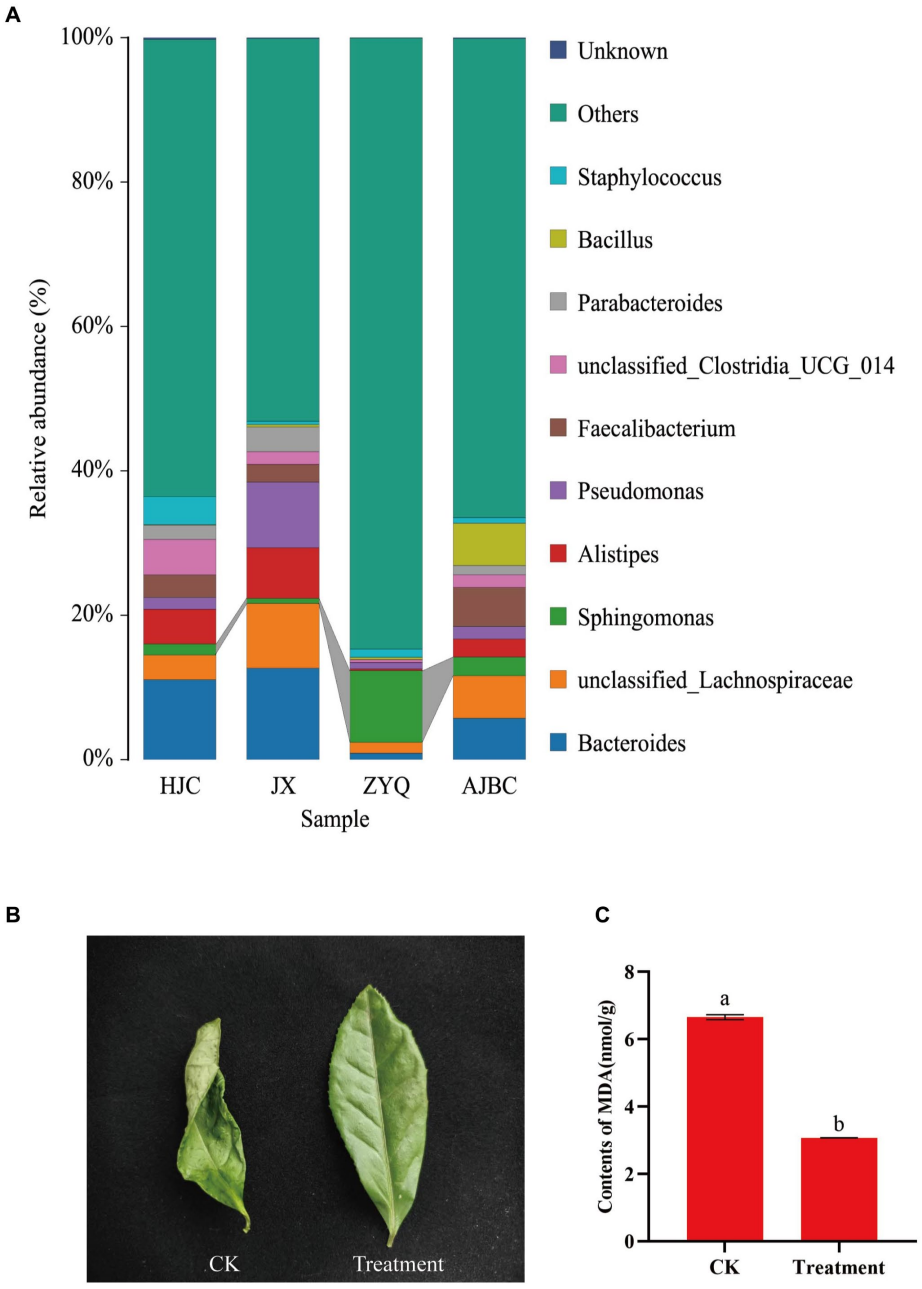
Screening of drought-resistant microorganisms in tea plants with higher levels of tea polyphenols. **(A)** Species stacking diagram with the top 10 bacterial genera for each treatment, and other bacterial genera categorized as “other.” **(B)** Photographs of leaves in the sterilized water spraying (CK) and *Sphingomonas* fermentation liquid spraying (Treatment). **(C)** MDA content between the two treatments. Different letters indicate significant differences (*p* < 0.05, T-test).

In addition to disease-resistant microorganisms, the investigation into other potentially beneficial microorganisms revealed a significant enrichment of *Sphingomonas* in tea plants with higher levels of tea polyphenols, ZYQ (Figure 8A). Following this outcome, drought experiments on tea seedlings were conducted utilizing *Sphingomonas* fermentation liquid., where tea seedlings treated with *Sphingomonas* fermentation liquid showed significantly higher drought resistance (Figures 8B,C; Supplementary Figure S1). Despite wilting in both the treatment and control groups, the treatment group exhibited a lesser degree of wilting. Moreover, the MDA content in the leaves of the treatment group was significantly lower than that in the control group. Interestingly, the relative abundance of *Sphingomonas* also exhibits a significant positive correlation with tea polyphenol contents, similarly to the aforementioned *Methylobacterium* (Supplementary Table S8).

## 4 Discussion

### 4.1 The host specificity of the microbial community associated with tea plants

The study of plant varieties and their interaction with microbial community has garnered significant attention due to its implications for sustainable agriculture and plant health. In the intricate web of ecological interactions, the specificity and variability of microbial community across different ecological niches present an intriguing area of study.

Subsequent sequencing and analyses of rhizosphere microorganisms and leaf endophytes revealed slight differences in *α*-diversity between species and ecological sites within the same environment (Figure 2). In this analyses, which examines both the diversity and abundance of the microbial community within the leaf endophytes and rhizosphere microbes, indicated no substantial differences among the tea varieties within the same ecological niches, overall. Covering both bacterial and fungal community. This finding suggests that the species richness and evenness of the microbial community at a given location are minimally influenced by the tea varieties. Therefore, we propose that the “space” available for microbial growth at the same ecological site is relatively consistent across different tea varieties.

However, the Venn diagrams and *β*-analyses has illuminated that varieties in plant species precipitate distinct microbial community within identical ecological niches (Figures 3, 4), including both the rhizosphere and the leaf interior. The intricate symbiosis between rhizosphere microorganisms and plants is well-documented (Enagbonma et al., 2023), with evidence from maize (Kong et al., 2020), and olive (Argyri et al., 2020) demonstrating that varietal changes significantly impact rhizosphere microorganisms by releasing distinct plant signals into the rhizosphere (Xu et al., 2018). This phenomenon underscores the importance of plant varieties in influencing rhizosphere microorganisms (Zhang et al., 2022). A similar pattern is observed in leaf endophytes, where a more pronounced host specificity is evident, as demonstrated by their more number of unique OTUs and distinct separation along the NMDS axis 1. This suggests that, compared to the rhizosphere of tea plants, there exist more intricate mechanisms within the tea leaves for constructing endophytic microbial community, such as microorganism-associated molecular patterns (MAMPs; Han, 2019). This pattern suggests that microbial host specificity is more pronounced in the above-ground parts of plants, including tea plants, a trend corroborated by other studies (Trivedi et al., 2020). This study emphasizes the differentials of microbial community across different varieties when grown in the same ecological niche, and also indicates the presence of a more complex microbial community assembly mechanism within the tea plant interior.

Interestingly, the endophytic microbial community of ZYQ exhibits a host specificity higher than that of the other three tea plant varieties. This is evidenced not only by the highest number of unique OTUs within ZYQ’s endophytic microbial community but also by its separation along the NMDS axis 1 from the other three tea plant varieties. Overall, these findings contribute to our understanding of plant-microbe interactions and the specificity of higher levels of tea polyphenols within the microbial community of the tea plant.

### 4.2 The across-ecological niche connections of the microbial community associated with tea plants

While the variability of microbial sources in the aboveground parts of plants is recognized (Vorholt, 2012), the soil’s microorganisms play a pivotal role in establishing plant microbial community (Coleman-Derr et al., 2016; Hamonts et al., 2018). This emphasizes the complex interactions between rhizosphere and leaf microbes (Hacquard and Schadt, 2015), highlighting the necessity of studying these interactions for a comprehensive understanding of plant microbial community dynamics. However, despite extensive research on individual plant loci (Tian et al., 2020), a holistic ecosystem-level understanding remains elusive. Our analyses of leaf endophytes alongside rhizosphere microorganisms in a co-occurrence network revealed that bacteria exhibit more complex and tighter connections between these ecological niches compared to fungi (Figure 4; Supplementary Tables S2–S5), This suggests that soil is the primary source of endophytic bacteria in tea plant leaves, while leaf endophytic fungi may originate from other sources (Hamonts et al., 2018). Further analyses of the nodes in the network reveals that, *Bacillus* has relatively high degree in the bacterial co-occurrence networks of the tea plants with higher tea polyphenol levels. There is ample evidence demonstrating the ability of *Bacillus* spp. to degrade phenolic compounds (Li et al., 2019), Considering the primary components of tea polyphenols, catechins, such as Epicatechin (EC), Epicatechin Gallate (ECG), Epigallocatechin (EGC), and Epigallocatechin Gallate (EGCG), they bear a striking resemblance in chemical structure to phenolic compounds, we speculated that *Bacillus* may degrade catechins in tea plants, thereby providing nutrients for other bacteria. Therefore, *Bacillus* may play a crucial role in shaping the bacterial community across-ecological niche within tea plants, constituting a core microbiota component therein (Astudillo-García et al., 2017).

As previously mentioned, we have devoted additional attention to the co-occurrence network of ZYQ. Our focus on tea plants with higher levels of tea polyphenols ZYQ revealed that these plants form the most symbiotic edges and highest connectivity between endophytic and rhizosphere bacteria, it is evidenced that tea plants with higher levels of tea polyphenols can reap greater benefits from favorable soil bacterial community, such as recruiting more beneficial endophytic bacteria from them, rather than solely absorbing additional nutrients (Bk et al., 2004). Moreover, we have discovered that *Methylobacterium*, *Sphingomonas*, and *Bacillus* play a more pivotal role in shaping the bacterial community across-ecological niche within ZYQ. It is well established that *Methylobacterium* and *Sphingomonas* are core microbiota in crops such as barley, rice, sugarcane, grapes, citrus, and soybeans. Their significance to microbial community is self-evident (Trivedi et al., 2020). Moreover, we have found that *Bacillus* also plays a significant role in shaping the cross-ecological niche bacterial community in ZYQ. Certain auxotrophic bacterial strains may exhibit superior host adaptability compared to their wild-type counterparts (Liu et al., 2018), these auxotrophic bacterial strains can meet their nutritional requirements through interactions among microorganisms, for instance, *B. subtilis*, a bacterial species within the genus *Bacillus*, can fulfill the thiamine auxotrophic requirement of the beneficial endophytic fungus *Serendipita indica*. Based on the findings above, we speculate that *Bacillus* within ZYQ may similarly contribute to the assembly of cross-ecosystem bacterial community, aiding tea plants in recruiting more distinct bacteria from the soil.

### 4.3 Tea polyphenols represent a potential influencing factor on beneficial endophytic microbial communities in tea plants

An initial examination of their tea polyphenol contents revealed notable variations among the varieties (Figure 1; Supplementary Table S1), despite being grown in identical environmental conditions. This observation underscores the inherent differences in tea polyphenol contents among tea plant varieties. It becomes imperative to prioritize the study of tea polyphenol contents. This is crucial for understanding the differences among tea plants. In this context, the tea plants with tea polyphenol contents exceeding 20% are considered to possess higher levels of these compounds. In this study, ZYQ was designated as a variety with higher levels of tea polyphenols for further analyses.

In the realm of agricultural science, the exploration of beneficial microorganisms and their impact on plant health and productivity has garnered significant attention (Suman et al., 2022). The presence of beneficial microorganisms, such as *Bacillus velezensis* and *Vibrio alginolyticus*, has been shown to bolster plant resistance against pathogens and environmental stresses, highlighting their potential in sustainable agriculture (Yuan et al., 2012; Stringlis et al., 2018; Ali et al., 2023; Ma et al., 2023). These findings are particularly relevant in the context of agricultural losses caused by phytopathogenic fungi, emphasizing the importance of exploring microbial avenues for disease suppression (Peng et al., 2021). However, the exploration of these microorganisms within the context of tea plants, especially in relation to tea polyphenol levels, remains relatively uncharted territory.

Existing research indicates that metabolites with specific characteristics of tea plants, such as L-theanine, participate in shaping microbiome assembly (Xie et al., 2022). Consequently, despite the potential absence of direct evidence, given the substantial biological activities attributed to tea polyphenols (Yuan et al., 2023), including their contribution to disease resistance(Yang and Zhang, 2019) and drought tolerance (Shao et al., 2023), we hypothesize that tea polyphenols is conducive to enriching beneficial microorganisms, leading to subsequent investigations on leaf endophytes and rhizosphere microorganisms. The leaf endophytes and rhizosphere microorganisms of ZYQ will be the focus of intensive analyses. In our research, we observed that the leaf microbiota of ZYQ exhibited a distinctive profile compared to the other three varieties. Moreover, the interplay between rhizosphere and leaf bacterial community appeared to be more complicated and closer. These findings substantiated our hypothesis and leaded to the subsequent analyses.

We conducted an analyses using LEfSe on the OTUs data, which revealed a significantly higher number of dominant microorganisms in ZYQ (Figure 6). Notably, the analyses indicated a greater enrichment of dominant bacteria over fungi in these plants, suggesting that bacteria should be the focal point of future research endeavors. This observation is similarly supported by the analyses of topological properties of nodes in the co-occurrence network.

Further analyses through FUNGuild prediction highlighted a reduced presence of phytopathogenic fungi in tea plants with higher levels of tea polyphenols (Figure 7A; Supplementary Table S6). While this results potentially due to the increased survival stress induced by higher tea polyphenols, we speculate that the enriched beneficial microbes in ZYQ may also contribute to pathogen resistance. To explore this hypothesis further, we examined the correlation between the abundance of the pathogen *Colletotrichum* (Jeyaraj et al., 2023) and the beneficial bacterium *Methylobacterium* (Zhang et al., 2021) uncovering a significant negative correlation (Figures 7B,C; Supplementary Table S7). This suggests that *Methylobacterium*, particularly enriched in higher levels of tea polyphenols tea plants (Liu et al., 2023), may play a crucial role in pathogen resistance.

Beyond disease resistance, our investigation also shed light on the potential advantages of beneficial microorganisms in other aspects of plant health. The genus *Sphingomonas*, known for its role in enhancing plant growth under various stresses (Asaf et al., 2017, 2020), showed a significant increase in abundance in higher levels of tea polyphenols tea plants ZYQ. Previous research indicates that MDA serves as a commonly used indicator for assessing the degree of oxidative stress, reflecting the extent of lipid peroxidation in plants (Janero, 1990). Therefore, MDA content is frequently utilized as a parameter in studies of plant senescence physiology and resistance physiology (Dastogeer et al., 2022). MDA levels provide insights into the degree of membrane lipid peroxidation, thereby indirectly assessing the extent of membrane damage and the resilience of plants to stress. Therefore, this experiment chose to characterize the resistance of tea plants under different treatments using MDA levels. The results indicate that, even under the same stress conditions, tea leaves treated with bacterial solution showed significantly lower MDA content compared to the control group (Figure 8; Supplementary Figure S1). This suggests that Sphingomonas benefits the drought resistance of tea plants. This finding suggests a potential contribution to improved drought tolerance, supported by experiments demonstrating the efficacy of *Sphingomonas* fermentation liquid in alleviating drought stress in tea seedling.

This study provides insights into the role of beneficial microorganisms in tea plants with higher levels of tea polyphenols, highlighting the importance of bacteria, particularly *Methylobacterium*, in pathogen resistance and the potential benefits of *Sphingomonas* in enhancing drought tolerance. These findings underscore the significance of exploring the microbial community associated with tea plants, offering promising avenues for future research aimed at harnessing the potential of beneficial microorganisms in agriculture.

## 5 Conclusion

This study has elucidated the intricate relationships within the microbial communities among different tea varieties, highlighted is the host specificity of tea plants in shaping the structures of microbial community, and the host specificity becomes more pronounced within endophytes residing in tea plant leaves. Our findings underscore the complexity and interconnectedness of the bacterial community within tea plants, which surpasses that of the fungal community across both the leaves and the rhizosphere, it indicated that bacteria in the soil serve as significant contributors to the bacterial composition within tea leaves. This complexity suggests a sophisticated bacterial ecosystem intrinsic to the tea plants. The analysis of Venn diagrams, NMDS results, co-occurrence analyses and LEfSe analysis demonstrated that tea plants rich in tea polyphenols, such as ‘Zhuyeqi’, foster distinct microbial community. This distinction underscores the potential of the tea plants with higher levels of tea polyphenols as invaluable repositories of beneficial microbial resources. These beneficial bacteria enriched by the tea plants with higher levels of tea polyphenol are active participants in preparing the tea plants to face future challenges, including diseases and abiotic stresses.

## Data Availability

The data presented in the study are deposited in the BioProject database, accession number PRJNA1146958.
