# Supplementary material for: Microbial diversity across tea varieties and ecological niches: correlating tea polyphenol contents with stress resistance
Source: Front Microbiol. 2024 Aug 26;15:1439630. doi: 10.3389/fmicb.2024.1439630 (PMC11381266; doi:10.3389/fmicb.2024.1439630)
Supplement: Supplementary file 1 [file Data_Sheet_1.zip › Supplyment figure .docx]

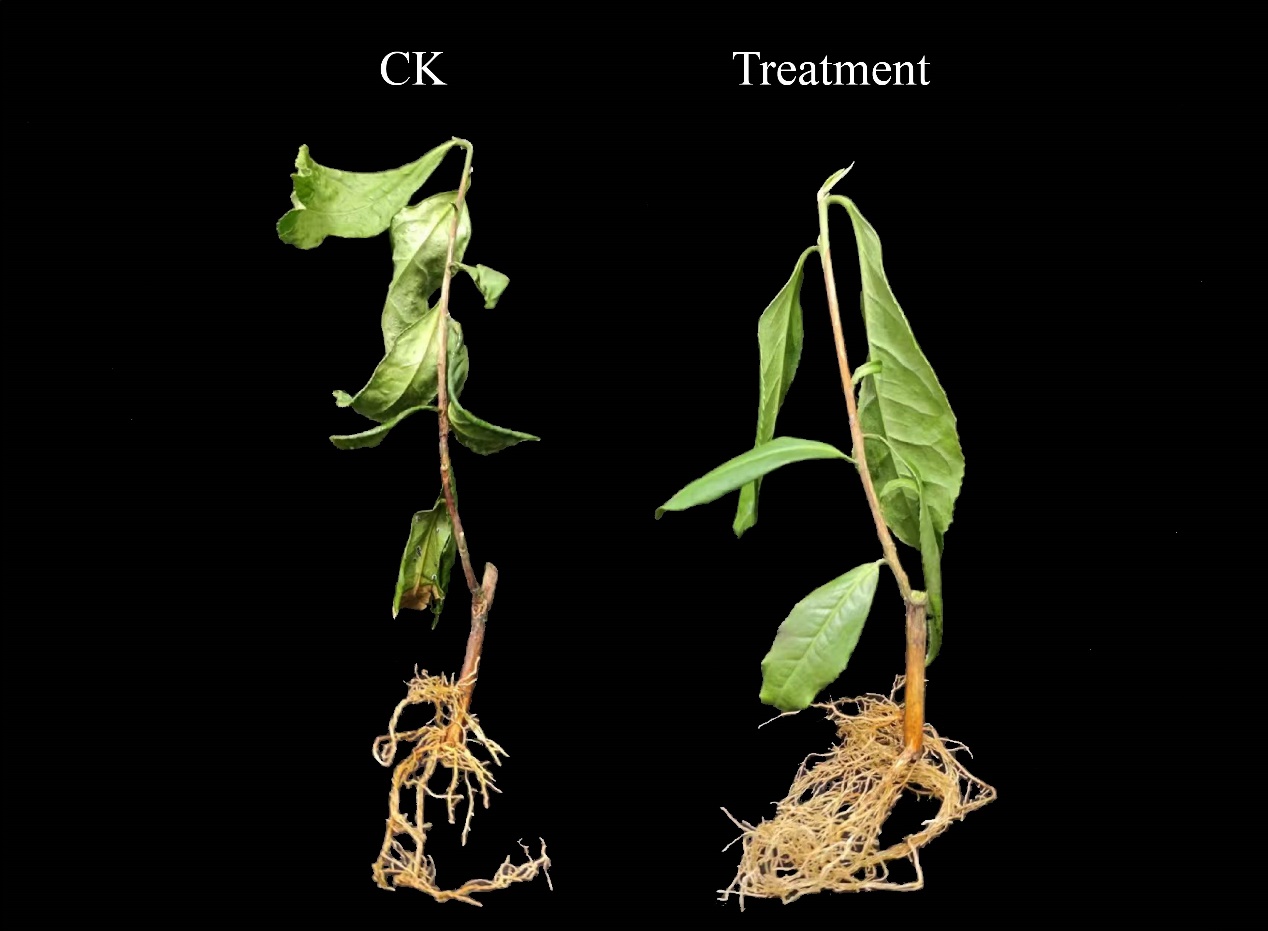


Fig S1. A comparison of the effects of sterilized water spraying(CK) and *Sphingomonas* fermentation liquid spraying(Treatment) on tea seedlings after drought treatment.
