# Supplementary material for: Microbial diversity across tea varieties and ecological niches: correlating tea polyphenol contents with stress resistance
Source: Front Microbiol. 2024 Aug 26;15:1439630. doi: 10.3389/fmicb.2024.1439630 (PMC11381266; doi:10.3389/fmicb.2024.1439630)
Supplement: Supplementary file 1 [file Data_Sheet_1.zip › Table S8.docx]

| Genus | R^2^ | P value | Equation |
| --- | --- | --- | --- |
| Sphingomonas | 0.6701 | <0.0001 | Y=0.01812*X-0.3126 |
| Methylobacterium | 0.7443 | <0.0001 | Y=0.00632*X-0.1120 |

Table S8: The Pearson correlation analysis results for *Sphingomonas* and *Methylobacterium* with tea polyphenol contents.

NOTE: X: tea polyphenol content; Y: relative abundance
